# Developing and evaluating a mobile driver fatigue detection network based on electroencephalograph signals

**DOI:** 10.1049/htl.2016.0053

**Published:** 2016-10-20

**Authors:** Jinghai Yin, Jianfeng Hu, Zhendong Mu

**Affiliations:** The Center of Collaboration and Innovation, Jiangxi University of Technology, Yao Lake University Park, Nanchang 330098, People's Republic of China

**Keywords:** electroencephalography, road traffic, cloud computing, fuzzy logic, entropy, support vector machines, accident prevention, medical signal detection, mobile driver fatigue detection network, electroencephalograph signals, traffic safety, middleware architecture, process unit, personal electroencephalography node, cloud server, android application, fuzzy entropy, support vector machine

## Abstract

The rapid development of driver fatigue detection technology indicates important significance of traffic safety. The authors’ main goals of this Letter are principally three: (i) A middleware architecture, defined as process unit (PU), which can communicate with personal electroencephalography (EEG) node (PEN) and cloud server (CS). The PU receives EEG signals from PEN, recognises the fatigue state of the driver, and transfer this information to CS. The CS sends notification messages to the surrounding vehicles. (ii) An android application for fatigue detection is built. The application can be used for the driver to detect the state of his/her fatigue based on EEG signals, and warn neighbourhood vehicles. (iii) The detection algorithm for driver fatigue is applied based on fuzzy entropy. The idea of 10-fold cross-validation and support vector machine are used for classified calculation. Experimental results show that the average accurate rate of detecting driver fatigue is about 95%, which implying that the algorithm is validity in detecting state of driver fatigue.

## 1 Introduction

Driver fatigue is receiving more and more attention in the traffic safety field, because it affects the driver's ability to make decision, slow down reaction time, decrease driver's attention, and contributes for increasing the number of accidents [1]. More and more researches show that driving accidents are largely related to fatigue [2, 3]. According to statistical results of the National Highway Transportation and Safety Administration (NHTSA), driver fatigue accounts for most crashes in the United States [4]. If driver fatigue can be detected, drivers will get useful information about their fatigue and so decrease the traffic accident [5].

Physical measures of driver fatigue are used, including fixed gaze, eye exposure duration, frontal face pose, blink frequency, and nodding frequency [6, 7]. Furthermore, numerous experiments show physiological signals can be applied to detect fatigue state [8], including electroencephalograph (EEG), electrooculography (EOG), electrocardiogram (ECG) and electromyogram (EMG). Electroencephalography (EEG) is a direct reflection of the brain's activity. Numerous brain and psychological studies have used EEG to study the neural activity underlying different emotional and psychological phenomena. Drivers have reduced levels of alertness when they are fatigued. This is accompanied by some consistently measurable changes in the EEG signals. EEG changes during driver fatigue can be utilised in the driver fatigue detection system [9–13].

In this work, we developed and evaluated a mobile driver fatigue detection network based on EEG signals.

## 2 System overview

**Fig. 1:**
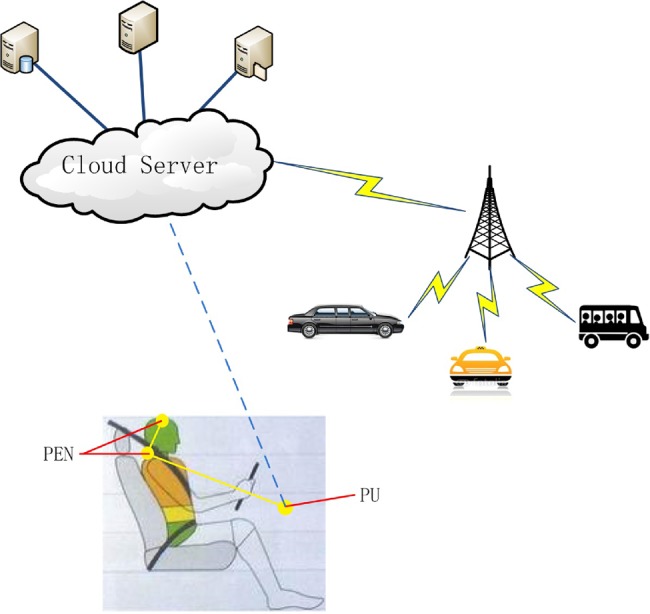
Proposed system

We propose a system (Fig. 1), where the EEG signals of driver are detected to avoid traffic accidents. EEG signals of driver are measured by personal EEG node (PEN), and sent to a process unit (PU) located in the vehicle. The PU can communicate with cloud server (CS).

In this system, the EEG signals transmitted by the PEN are processed in the PU to detect the driver's fatigue state. Since driver fatigue is a dangerous to vehicles around the driver, the surrounding vehicles should be warned for safety. If the driver is detected to be fatigue, messages are sent from the PU to CS.

**Fig. 2:**
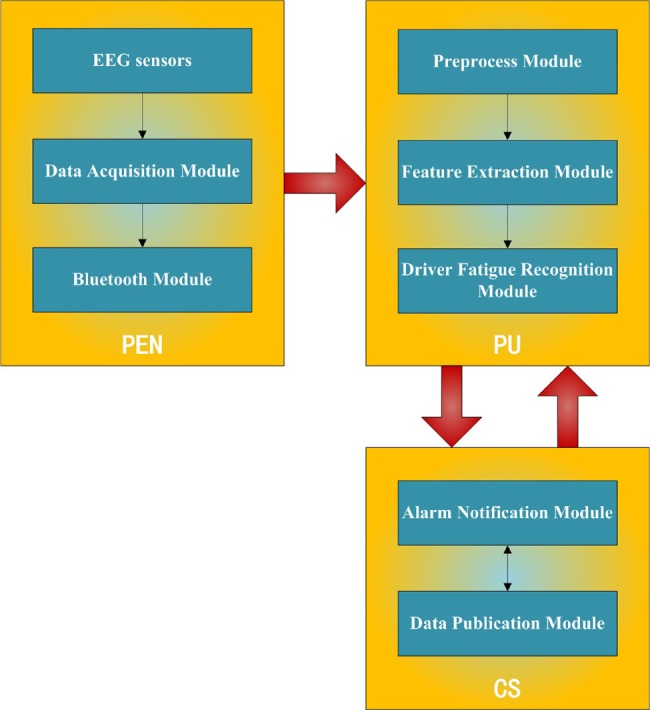
Integration architecture

**Fig. 3:**
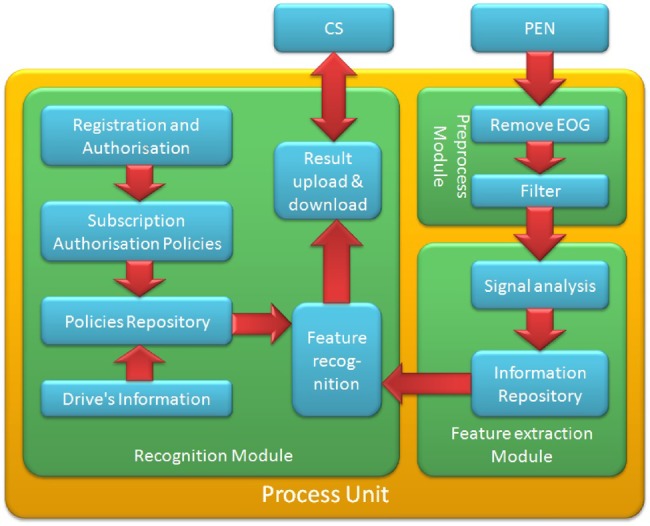
PU architecture

The proposed system consists of modular integration of PEN, PU and CS (Fig. 2). PEN consists of EEG sensors module, Data acquisition module and Bluetooth module, which measure EEG signals and transmitted it via Bluetooth module to PU. PU is the key unit of the system, which consists of preprocess module, feature extraction module and driver fatigue recognition module (Fig. 3). Preprocess module receives EEG signals from PEN, and the signals are filtered and artefact-removal. Features are extracted from EEG signals in feature extraction module. These features are determined by the driver fatigue recognition module to estimate if the driver is fatigue. CS consists of alarm notification module and data publication module, according to the CS, alarm notification module generates a notification message from the driver's fatigue data uploaded by PU. Then data publication module send notification messages to vehicle surround the driver if the driver is fatigue.

In Fig. 3, we can see that PU is composed of three modules, which are preprocess module, feature extraction module and recognition module. PU has an interface for the PEN and another interface for CS. EEG signals collected from the PEN are first sent to the preprocess module, where artefacts are removed. Then the signals are sent to the feature extraction module, where signal analysis and feature extraction are conducted. The features extracted from EEG signals are sent to the recognition module, and the detection results are uploaded to a CS. EEG data should be preprocessed and transmitted to CS due to driver fatigue. The registration module is responsible for the user to enter the application and authentication module is responsible for the identification of user's identity. The subscription and authorisation module is responsible for protecting the user's privacy.

## 3 Experimentation

**Fig. 4:**
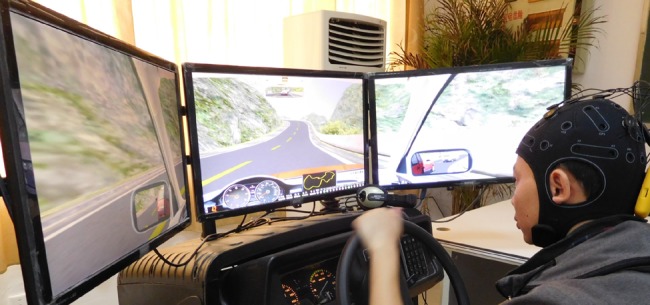
Subject in the simulated driving experiment

A driving simulator (Fig. 4) was used to detect the driver fatigue. In the experiment, subjects were seated on a soft chair without armrests in a quiet shielded room, watching a screen, and making appropriate responses according to the test criteria and the indication screen. The subjects can imitate all kinds of road in front of the computer, control the direction of the car through the steering wheel, and control the speed of the car through the accelerator and brake.

Twelve healthy subjects ranging in age from 22 to 30 years participated in the driving experiment. They had no physical obstacles and can complete the driving task successfully. The drivers were demanded to finish specific tasks and guarantee safe driving [14, 15]. Before the experiments, subjects were familiar with the operation of the driving simulator and the completion of the driving task. All other vehicles and road stimuli were removed, and subjects were required to maintain a driving speed between 60 and 80 km/h. EEG acquisition during two driving sessions (normal sessions and fatigue driving sessions) was performed using StarStim EEG recording system (NE, USA) as PEN, with a reference electrode placed on the right mastoid. Based on that the forehead EEG signal collects expediently, we used only two electrodes FP1 and FP2 as signal source according to international 10–20 system. The sampling rate was set at 1000 Hz. Band acquisition used 200 Hz low-pass, 0.05 Hz high-pass and 50 Hz notch filters.

## 4 PU implementation

We have developed a fatigue detection application based on Android system as PU [16–18]. The application can receive EEG signals from PEN, detect the state of driver fatigue and establish communication with CS.

In the driving process, PEN collects the driver's EEG signals and transmits to the Android application via a Bluetooth wireless connection. Once EEG signals are received by the application, the data is preprocessed, features are extracted and the fatigue state is analysed. A warning system is triggered due to driver fatigue, and notification messages will be sent.

A fuzzy function was used to measure the degree of similarity of vectors, rather than the two-valued function in the sample entropy-based algorithm, such that the calculated entropy values are continuous and smooth [19, 20]. The procedure for the fuzzy entropy-based algorithm is described in detail as follows:

- Assuming *X_i_* is EEG signals $\left\{{X\lpar i\rpar \colon \; 1 \le i \le N} \right\}$.
- The phase-space reconstruction is performed on *X_i_* according to the sequence order, and a set of *m*-dimensional vectors are obtained $\lpar m \le N - 2\rpar $. The reconstructed vector ${\bi Y}_i^m $ can be written as
  (1) $${\bi Y}_i^m = \left\{{X_i\comma \; X_{i + 1}\comma \; \ldots \comma \; X_{i + m - 1}} \right\}- {\rm MEAN}\eqno\lpar 1\rpar $$
  where $i = 1\comma \; 2\comma \; \ldots \comma \; N - m + 1$, and *MEAN* is the average value.
- $d_{ij}^m $ is defined as the maximum difference values between the corresponding elements of two vectors ${\bi Y}_i^m \, \, {\rm and}\, \, {\bi Y}_j^m $
  $$\eqalign{d_{ij}^m &= d\left[{{\bi Y}_i^m \comma \; {\bi Y}_j^m } \right] \cr & = \mathop {\max }\limits_{k \in \lpar 0\comma m - 1\rpar } \lcub \vert \lpar X_{i + k} - {\rm MEA}{\rm N}_i\rpar - \lpar X_{\,j + k} - {\rm MEA}{\rm N}_j\rpar \vert \rcub \cr \lpar i\comma \; j &= 1\sim N - m\comma \; j \ne i\rpar} \eqno\lpar 2\rpar $$
- The similarity degree $D_{ij}^m $ between two vectors ${\bi Y}_i^m \, \, {\rm and}\, \, {\bi Y}_j^m $ is defined as follows
  (3) $$D_{ij}^m {\rm = exp}\lpar \!\!-\! \lpar d_{ij}^m \rpar ^n{\rm /}r\rpar \eqno\lpar 3\rpar $$
  where *n* and *r* are the gradient and width of the exponential function, respectively.
- Define the function $\varphi ^m\lpar n\comma \; r\rpar $
  (4) $$\varphi ^m\lpar n\comma \; r\rpar = \displaystyle{1 \over {N - m}}\mathop \sum \limits_{i = 1}^{N - m} \left[{\displaystyle{1 \over {N - m - 1}}\mathop \sum \limits_{\,j = 1\comma j \ne i}^{N - m} D_{ij}^m } \right]\; \; \; \; \; \; \; \eqno\lpar 4\rpar $$
- The fuzzy entropy can be expressed as follows
  (5) $${\rm FuzzyEn}\lpar m\comma \; n\comma \; r\comma \; N\rpar = \ln \varphi ^m\left({n\comma \; r} \right)- \ln \varphi ^{m + 1}\left({n\comma \; r} \right)\; \; \; \eqno\lpar 5\rpar $$
  where *m* and *r* are the dimensions of phase space and similarity tolerance, respectively. Generally, too large of a similarity tolerance will lead to a loss of useful information. The larger the similarity tolerance, the more information may be missed. However, if the similarity tolerance is underestimated, the sensitivity to noise will be increased significantly. In this Letter, we set *m* = 2, *n* = 4, *r* = 0.2 × *SD*, where *SD* denotes the standard deviation of the time series.

Support vector machine (SVM) is a kind of machine learning method based on statistical learning theory, which maps the input vector into a high dimensional feature space (hyperplane) through the appropriate mapping. The hyperplane is classification plane. SVM was used as the classifier. In this work, the popular radial basis function was used as the kernel function. To verify the feasibility of the system, we used Matlab to calculate, analyse and validate with the collected EEG signal, including the training and testing of the samples.

## 5 Result

**Fig. 5:**
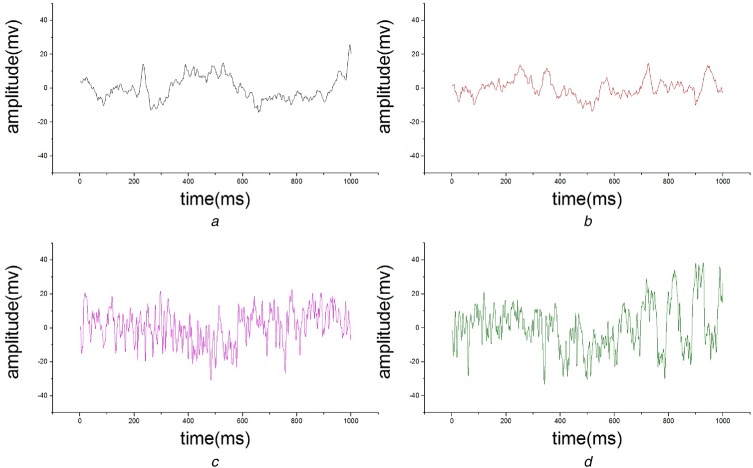
Sample EEG signals *a* and *b* For normal state from FP1 channel *c* and *d* For fatigue state from FP1 channel

In this work, the EEG signals under two different states (normal and fatigue) are considered. Fig. 5 shows sample EEG signals obtained from FP1 channel during different trials. As shown in Fig. 5, there is a significant difference between normal state and fatigue state of the EEG signal, however, it's hardly detect fatigue state only by this visual distinction. It must have some characteristic parameters, by which fatigue state can be detected properly and scientifically.

Therefore, in this Letter, we take entropy fuzzy as parameters for detection, i.e. by calculating value of fuzzy entropy of different electrodes of different trials, the fatigue state can be determined.

**Fig. 6:**
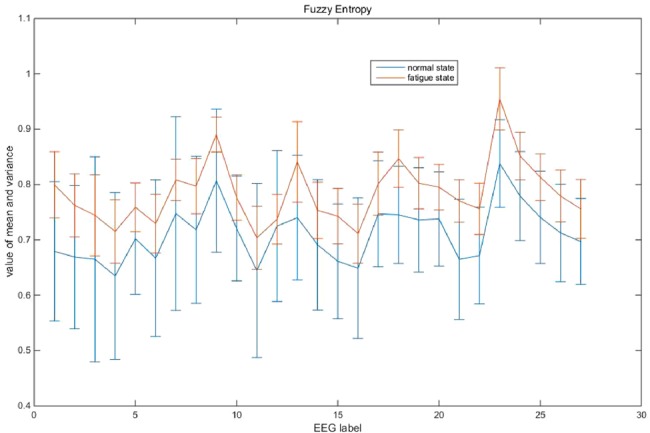
Comparison of fuzzy entropy value

Fig. 6 shows the fuzzy entropy characteristic of a subject, from which we can see the obvious difference of fuzzy entropy between the two states. The fuzzy entropy on fatigue state is obviously higher than that on normal state, so the fuzzy entropy is a preferable parameter to determine fatigue state.

One of the most common techniques to prove the stability of algorithm is replication of training by 10-fold cross-validation. Then 10-fold cross-validation was used to prove the consistency of algorithm and assess the classification accuracy, the mean values of test results are shown in Table 1. In recent years, several teams worked on this problem from using EEG brain signals to study driving fatigue detection and made a lot of research results as shown in Table 2, which implying that our result was far higher than other several classification methods.

**Table 1:** Test results given by10-fold cross-validation

No. of subject	Accuracy
1	0.95
2	0.96
3	0.97
4	0.98
5	0.94
6	0.96
7	0.97
8	0.95
9	0.93
10	0.97
11	0.94
12	0.99
MEAN ± VARIANCE	0.95 ± 0.017

**Table 2:** Performance comparison of the previous works

Author	Method	Accuracy, %
Xiongn *et al*. [21]	combined entropy	90
Kaur and Singh [22]	EMD	84.8
Correa *et al.* [23]	multimodal analysis	83.6
This paper	fuzzy entropy	95

**Fig. 7:**
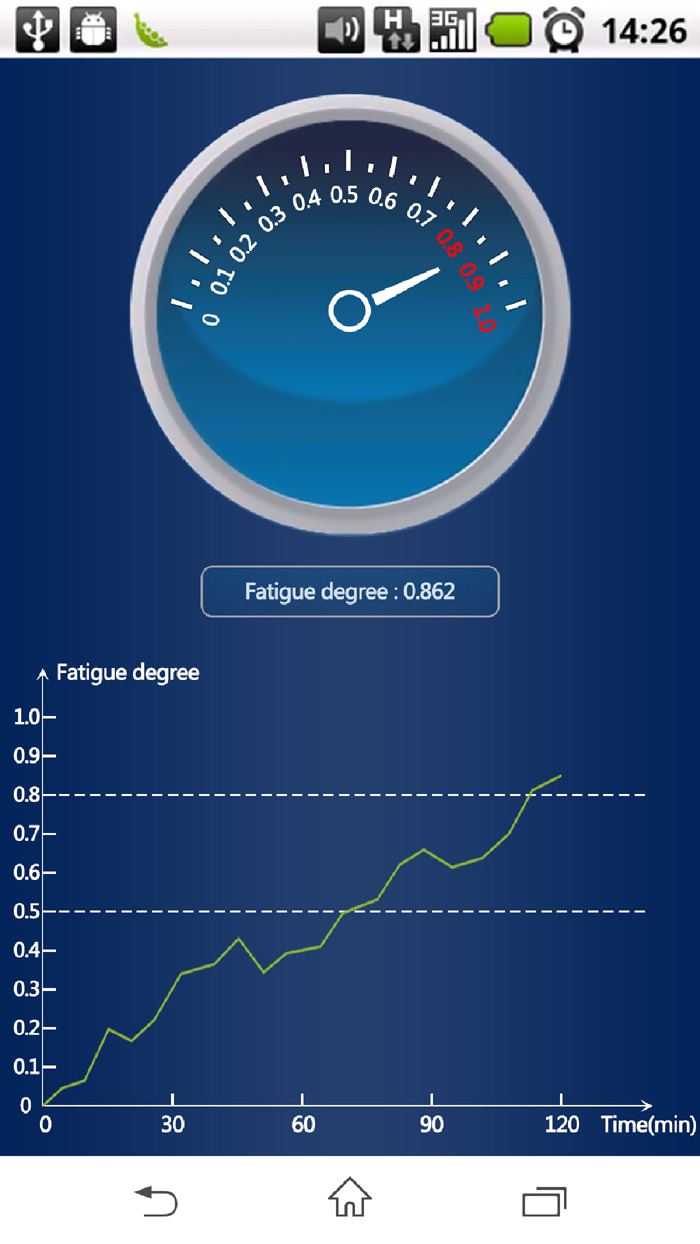
Android application

The interface of android application (Fig. 7) is composed of three parts. Above part is a pointer type display for fatigue level, where the scale varies from 0 to 1.0. Middle part is the exact value of the current fatigue level. The bottom part is the curve of the degree of fatigue change with time.

## 6 Discussion and conclusion

The system will face many challenges. First, because PEN maybe restrict drivers’ movement, how to keep the driver's comfort while wearing PEN is important. Safety belt is sometimes considered so uncomfortable that some drivers unlike wear it. A fewer number of sensors will be wore more comfortably. Second, the accuracy of the fatigue detection is also problematic. Detecting fatigue is especially difficult because of the characteristics of EEG signals. Third, how to protect the privacy of driver is a challenge. New and effective biometrics method should be applied for authentication and security [24, 25].

In this Letter, an architecture for the integration of PEN, PU and CS to detect driver fatigue has been established. Its main components and functions have been introduced. An Android application is applied to monitor the driver's fatigue state to avoid accident.
